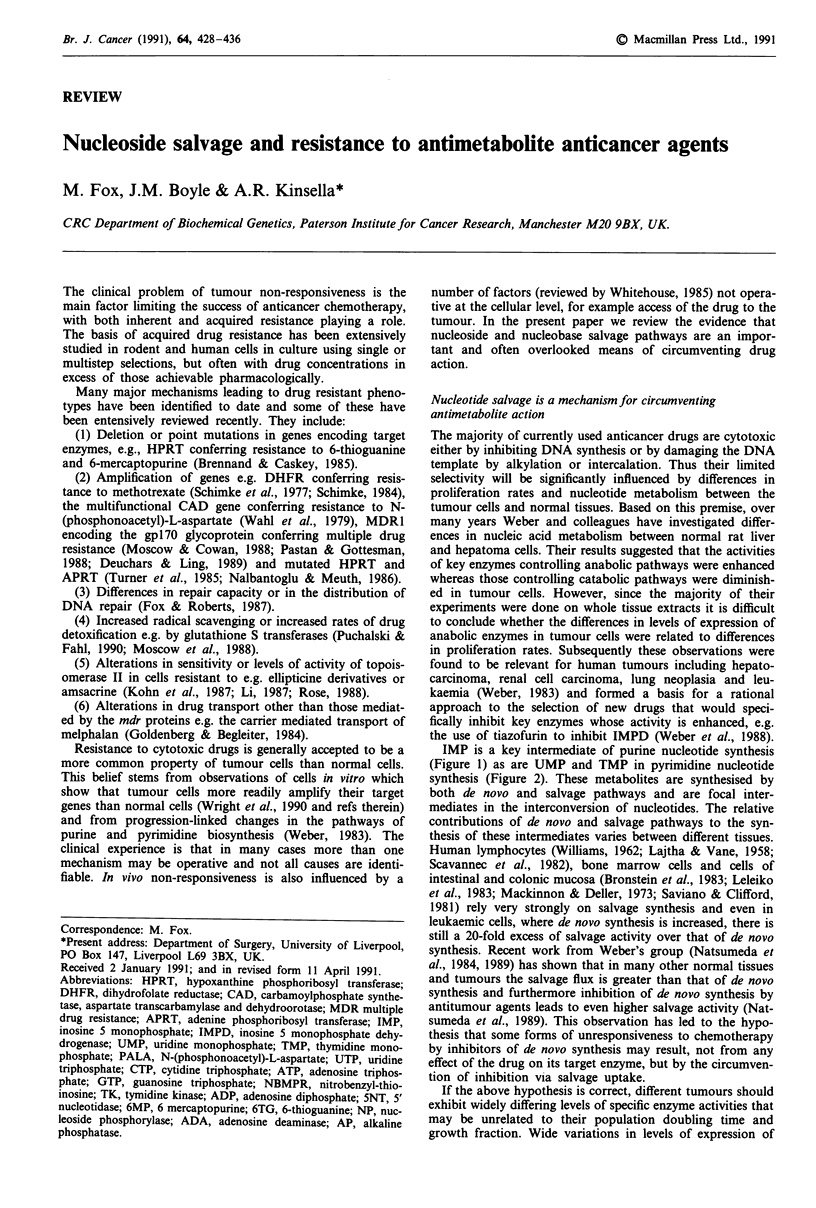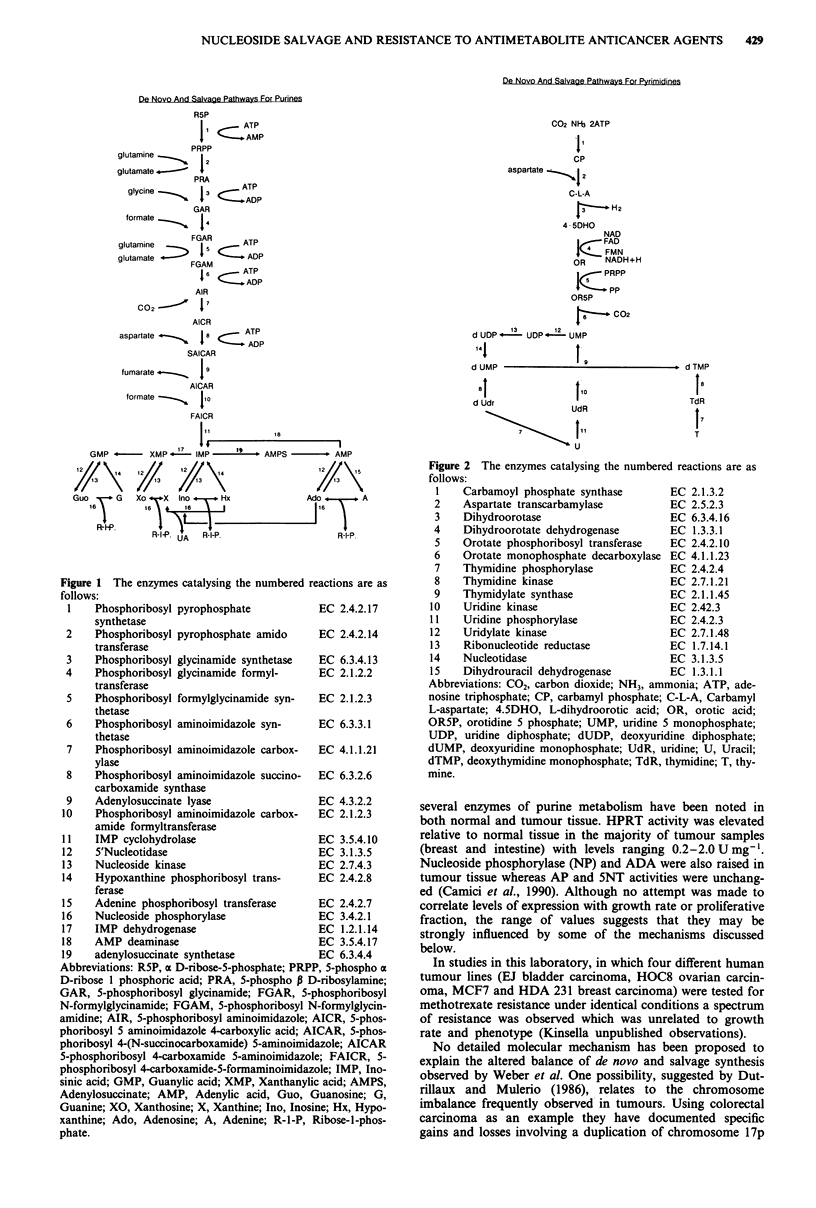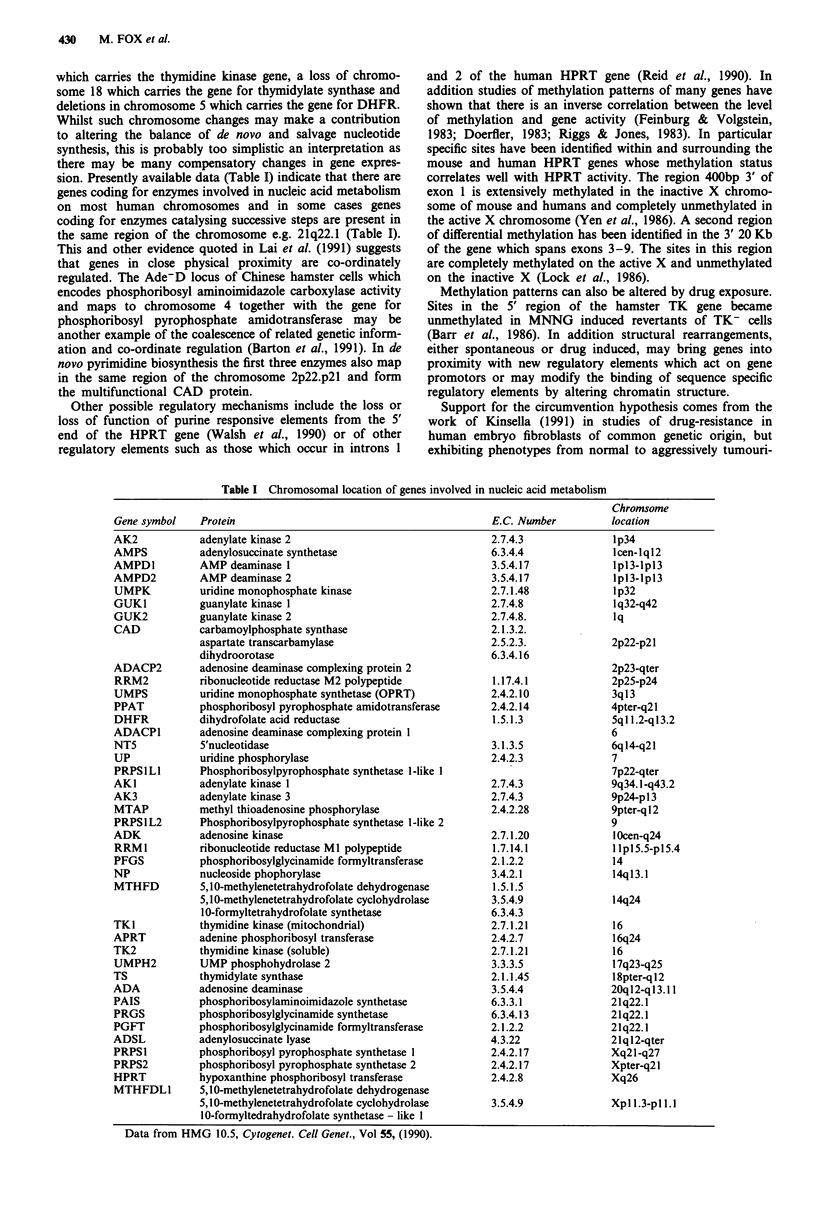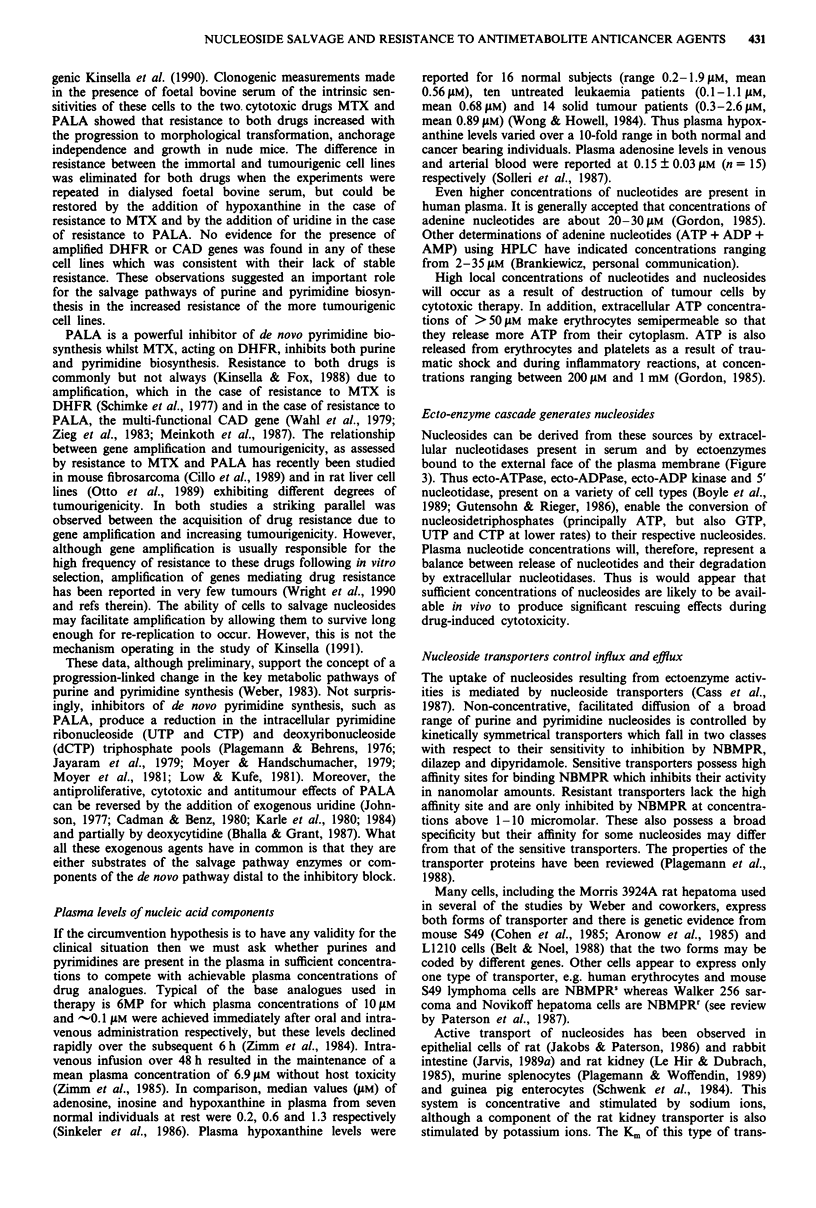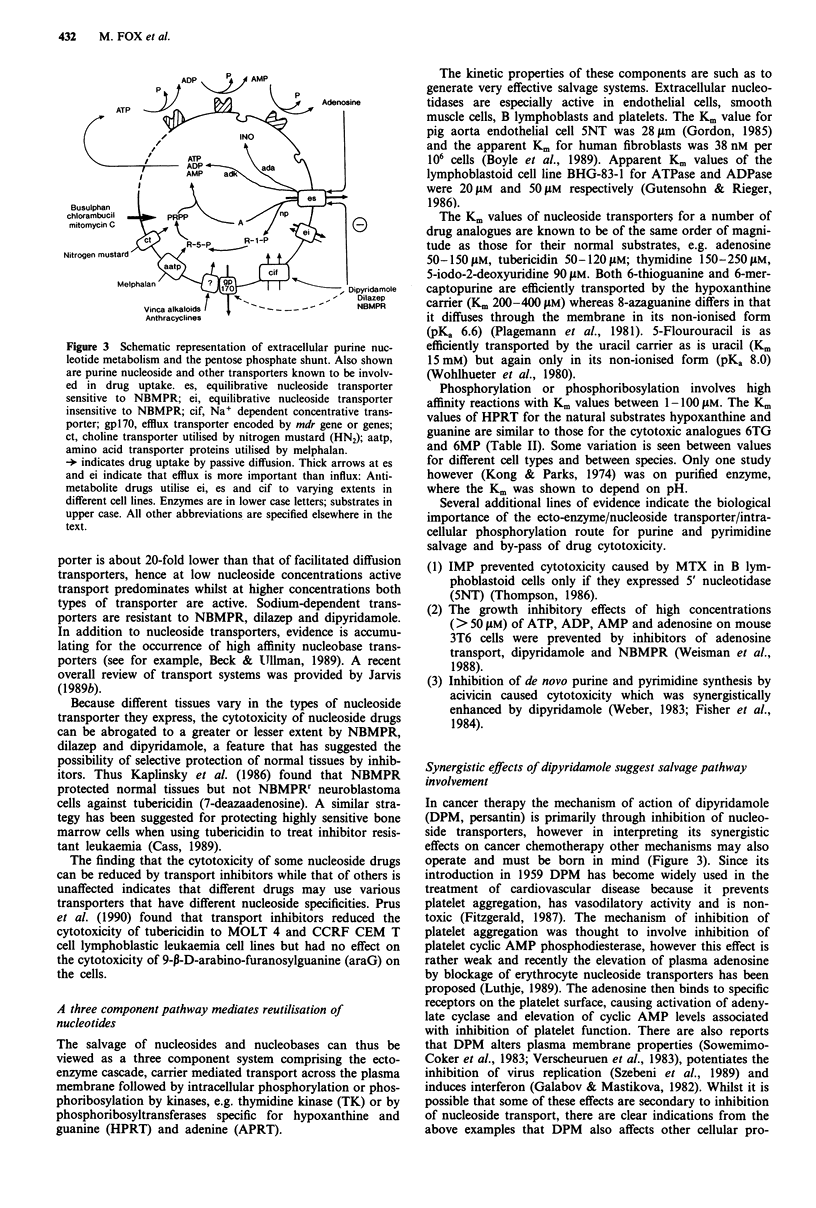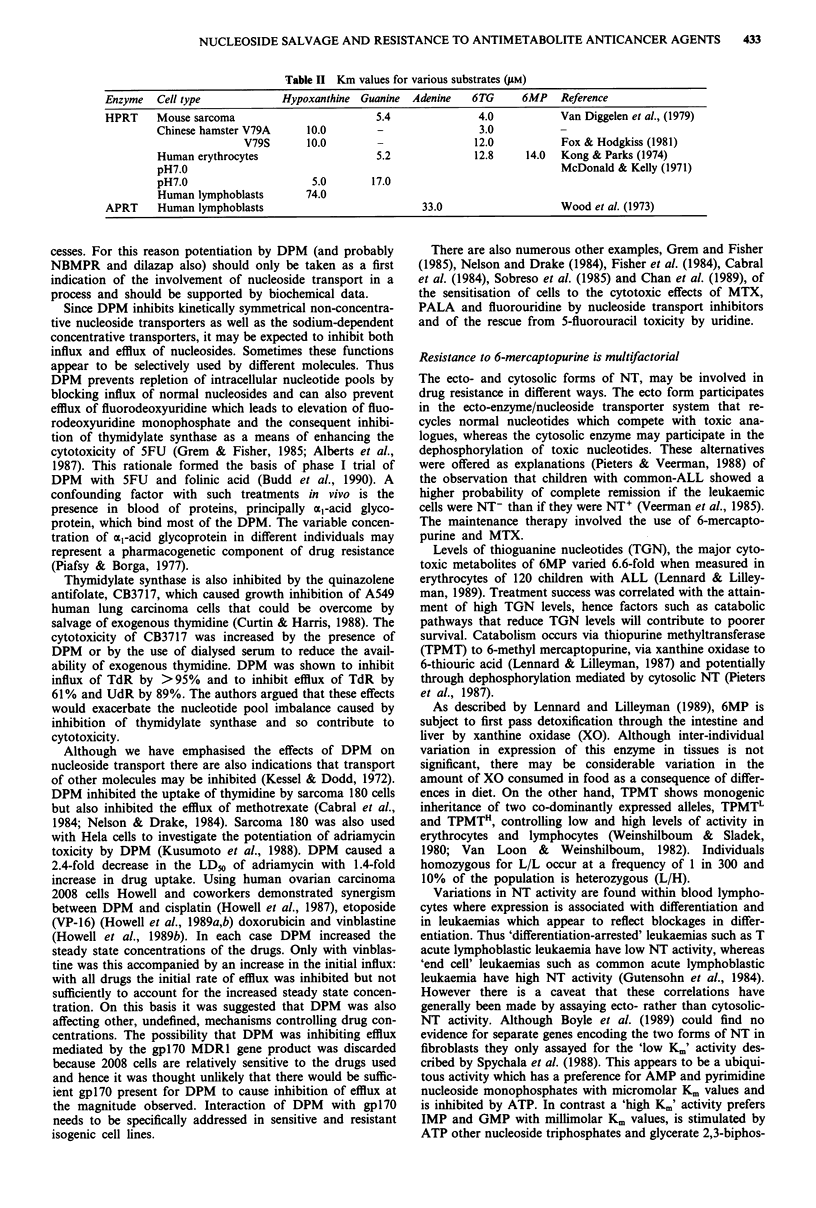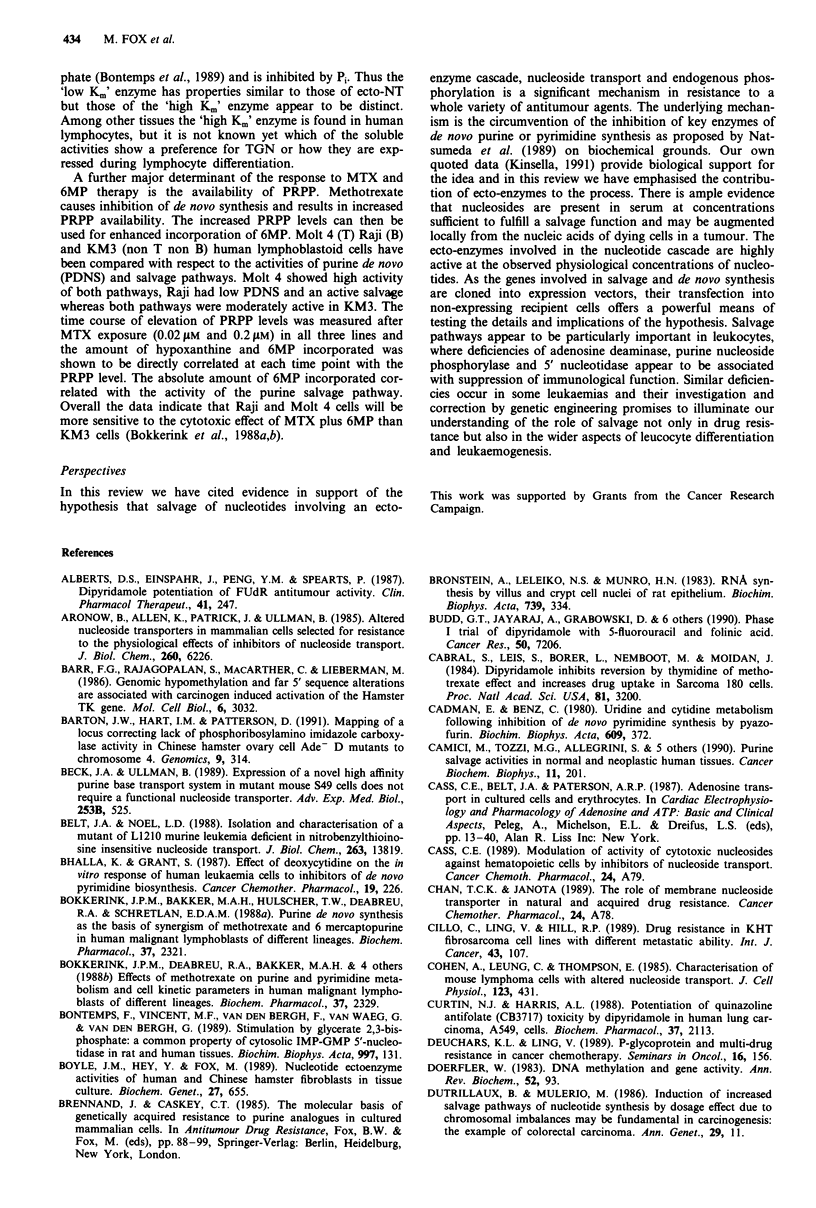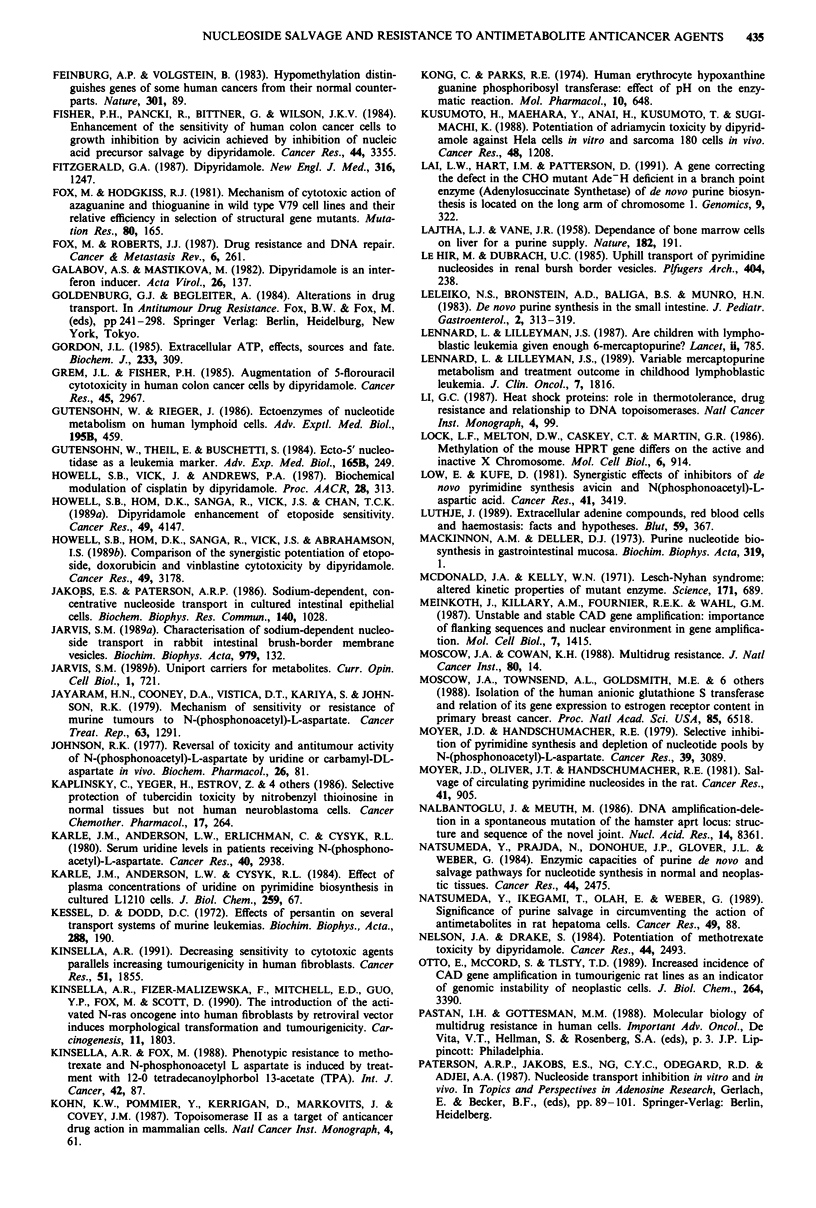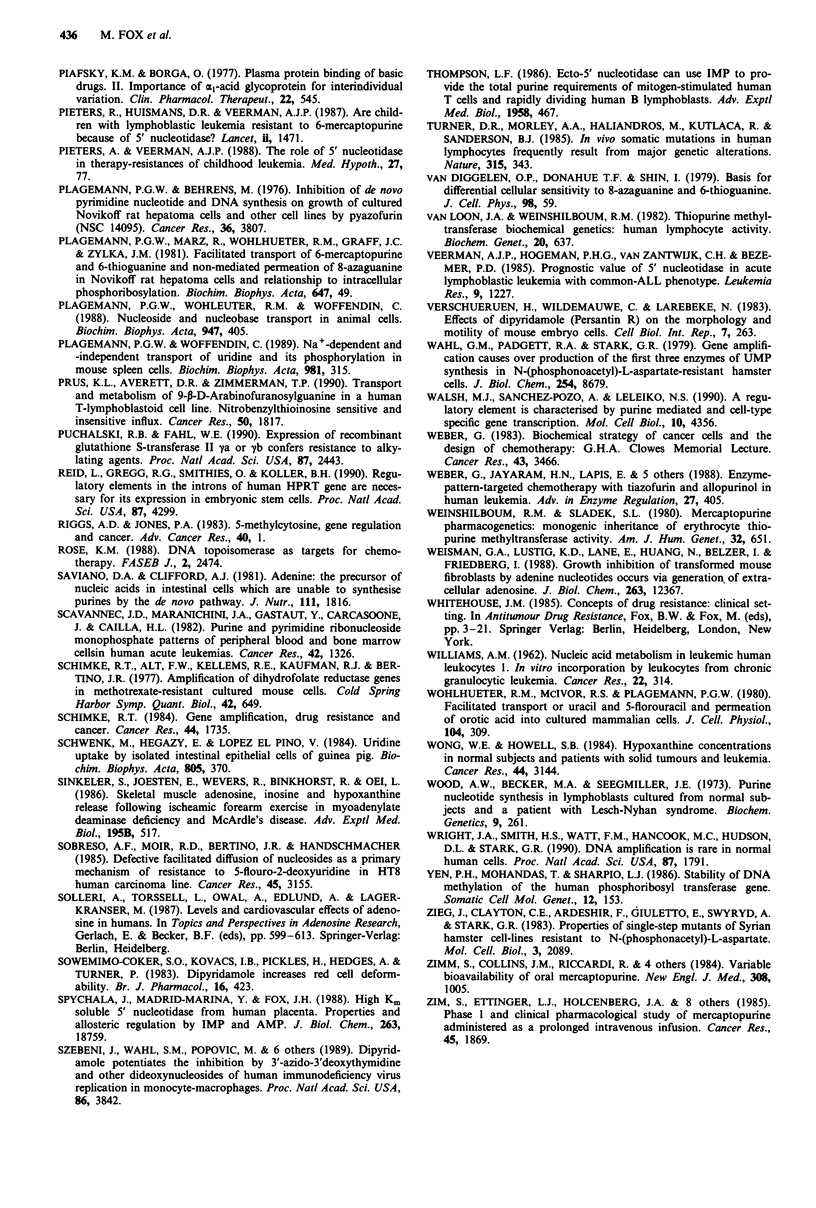# Nucleoside salvage and resistance to antimetabolite anticancer agents.

**DOI:** 10.1038/bjc.1991.327

**Published:** 1991-09

**Authors:** M. Fox, J. M. Boyle, A. R. Kinsella

**Affiliations:** CRC Department of Biochemical Genetics, Paterson Institute for Cancer Research, Manchester, UK.


Br. J. Cancer (1991), 64, 428-436 © Macmillan Press Ltd., 1991

REVIEW

### Nucleoside salvage and resistance to antimetabolite anticancer agents

M. Fox, J.M. Boyle & A.R. Kinsella*

*CRC Department of Biochemical Genetics, Paterson Institute for Cancer Research, Manchester M20 9BX, UK.*

The clinical problem of tumour non-responsiveness is the main factor limiting the success of anticancer chemotherapy, with both inherent and acquired resistance playing a role. The basis of acquired drug resistance has been extensively studied in rodent and human cells in culture using single or multistep selections, but often with drug concentrations in excess of those achievable pharmacologically.

Many major mechanisms leading to drug resistant phenotypes have been identified to date and some of these have been entensively reviewed recently. They include:

(1) Deletion or point mutations in genes encoding target enzymes, e.g., HPRT conferring resistance to 6-thioguanine and 6-mercaptopurine (Brennand & Caskey, 1985).

(2) Amplification of genes e.g. DHFR conferring resistance to methotrexate (Schimke *et al.*, 1977; Schimke, 1984), the multifunctional CAD gene conferring resistance to N-(phosphonoacetyl)-L-aspartate (Wahl *et al.*, 1979), MDR1 encoding the gp170 glycoprotein conferring multiple drug resistance (Moscow & Cowan, 1988; Pastan & Gottesman, 1988; Deuchars & Ling, 1989) and mutated HPRT and APRT (Turner *et al.*, 1985; Nalbantoglu & Meuth, 1986).

(3) Differences in repair capacity or in the distribution of DNA repair (Fox & Roberts, 1987).

(4) Increased radical scavenging or increased rates of drug detoxification e.g. by glutathione S transferases (Puchalski & Fahl, 1990; Moscow *et al.*, 1988).

(5) Alterations in sensitivity or levels of activity of topoisomerase II in cells resistant to e.g. ellipticine derivatives or amsacrine (Kohn *et al.*, 1987; Li, 1987; Rose, 1988).

(6) Alterations in drug transport other than those mediated by the *mdr* proteins e.g. the carrier mediated transport of melphalan (Goldenberg & Begleiter, 1984).

Resistance to cytotoxic drugs is generally accepted to be a more common property of tumour cells than normal cells. This belief stems from observations of cells *in vitro* which show that tumour cells more readily amplify their target genes than normal cells (Wright *et al.*, 1990 and refs therein) and from progression-linked changes in the pathways of purine and pyrimidine biosynthesis (Weber, 1983). The clinical experience is that in many cases more than one mechanism may be operative and not all causes are identifiable. *In vivo* non-responsiveness is also influenced by a number of factors (reviewed by Whitehouse, 1985) not operative at the cellular level, for example access of the drug to the tumour. In the present paper we review the evidence that nucleoside and nucleobase salvage pathways are an important and often overlooked means of circumventing drug action.

*Nucleotide salvage is a mechanism for circumventing antimetabolite action*

The majority of currently used anticancer drugs are cytotoxic either by inhibiting DNA synthesis or by damaging the DNA template by alkylation or intercalation. Thus their limited selectivity will be significantly influenced by differences in proliferation rates and nucleotide metabolism between the tumour cells and normal tissues. Based on this premise, over many years Weber and colleagues have investigated differences in nucleic acid metabolism between normal rat liver and hepatoma cells. Their results suggested that the activities of key enzymes controlling anabolic pathways were enhanced whereas those controlling catabolic pathways were diminished in tumour cells. However, since the majority of their experiments were done on whole tissue extracts it is difficult to conclude whether the differences in levels of expression of anabolic enzymes in tumour cells were related to differences in proliferation rates. Subsequently these observations were found to be relevant for human tumours including hepatocarcinoma, renal cell carcinoma, lung neoplasia and leukaemia (Weber, 1983) and formed a basis for a rational approach to the selection of new drugs that would specifically inhibit key enzymes whose activity is enhanced, e.g. the use of tiazofurin to inhibit IMPD (Weber *et al.*, 1988).

IMP is a key intermediate of purine nucleotide synthesis (Figure 1) as are UMP and TMP in pyrimidine nucleotide synthesis (Figure 2). These metabolites are synthesised by both *de novo* and salvage pathways and are focal intermediates in the interconversion of nucleotides. The relative contributions of *de novo* and salvage pathways to the synthesis of these intermediates varies between different tissues. Human lymphocytes (Williams, 1962; Lajtha & Vane, 1958; Scavannec *et al.*, 1982), bone marrow cells and cells of intestinal and colonic mucosa (Bronstein *et al.*, 1983; Leleiko *et al.*, 1983; Mackinnon & Deller, 1973; Saviano & Clifford, 1981) rely very strongly on salvage synthesis and even in leukaemic cells, where *de novo* synthesis is increased, there is still a 20-fold excess of salvage activity over that of *de novo* synthesis. Recent work from Weber's group (Natsumeda *et al.*, 1984, 1989) has shown that in many other normal tissues and tumours the salvage flux is greater than that of *de novo* synthesis and furthermore inhibition of *de novo* synthesis by antitumour agents leads to even higher salvage activity (Natsumeda *et al.*, 1989). This observation has led to the hypothesis that some forms of unresponsiveness to chemotherapy by inhibitors of *de novo* synthesis may result, not from any effect of the drug on its target enzyme, but by the circumvention of inhibition via salvage uptake.

If the above hypothesis is correct, different tumours should exhibit widely differing levels of specific enzyme activities that may be unrelated to their population doubling time and growth fraction. Wide variations in levels of expression of

Correspondence: M. Fox.
*Present address: Department of Surgery, University of Liverpool, PO Box 147, Liverpool L69 3BX, UK.
Received 2 January 1991; and in revised form 11 April 1991.
Abbreviations: HPRT, hypoxanthine phosphoribosyl transferase; DHFR, dihydrofolate reductase; CAD, carbamoylphosphate synthetase, aspartate transcarbamylase and dehydroorotase; MDR multiple drug resistance; APRT, adenine phosphoribosyl transferase; IMP, inosine 5 monophosphate; IMPD, inosine 5 monophosphate dehydrogenase; UMP, uridine monophosphate; TMP, thymidine monophosphate; PALA, N-(phosphonoacetyl)-L-aspartate; UTP, uridine triphosphate; CTP, cytidine triphosphate; ATP, adenosine triphosphate; GTP, guanosine triphosphate; NBMPR, nitrobenzyl-thioinosine; TK, tymidine kinase; ADP, adenosine diphosphate; 5NT, 5′ nucleotidase; 6MP, 6 mercaptopurine; 6TG, 6-thioguanine; NP, nucleoside phosphorylase; ADA, adenosine deaminase; AP, alkaline phosphatase.

**Figure 1** The enzymes catalysing the numbered reactions are as follows:

| | | |
|---|---|---|
| 1 | Phosphoribosyl pyrophosphate synthetase | EC 2.4.2.17 |
| 2 | Phosphoribosyl pyrophosphate amido transferase | EC 2.4.2.14 |
| 3 | Phosphoribosyl glycinamide synthetase | EC 6.3.4.13 |
| 4 | Phosphoribosyl glycinamide formyl-transferase | EC 2.1.2.2 |
| 5 | Phosphoribosyl formylglycinamide synthetase | EC 2.1.2.3 |
| 6 | Phosphoribosyl aminoimidazole synthetase | EC 6.3.3.1 |
| 7 | Phosphoribosyl aminoimidazole carboxylase | EC 4.1.1.21 |
| 8 | Phosphoribosyl aminoimidazole succino-carboxamide synthase | EC 6.3.2.6 |
| 9 | Adenylosuccinate lyase | EC 4.3.2.2 |
| 10 | Phosphoribosyl aminoimidazole carboxamide formyltransferase | EC 2.1.2.3 |
| 11 | IMP cyclohydrolase | EC 3.5.4.10 |
| 12 | 5'Nucleotidase | EC 3.1.3.5 |
| 13 | Nucleoside kinase | EC 2.7.4.3 |
| 14 | Hypoxanthine phosphoribosyl transferase | EC 2.4.2.8 |
| 15 | Adenine phosphoribosyl transferase | EC 2.4.2.7 |
| 16 | Nucleoside phosphorylase | EC 3.4.2.1 |
| 17 | IMP dehydrogenase | EC 1.2.1.14 |
| 18 | AMP deaminase | EC 3.5.4.17 |
| 19 | adenylosuccinate synthetase | EC 6.3.4.4 |

Abbreviations: R5P, α D-ribose-5-phosphate; PRPP, 5-phospho α D-ribose 1 phosphoric acid; PRA, 5-phospho β D-ribosylamine; GAR, 5-phosphoribosyl glycinamide; FGAR, 5-phosphoribosyl N-formylglycinamide; FGAM, 5-phosphoribosyl N-formylglycinamidine; AIR, 5-phosphoribosyl aminoimidazole; AICR, 5-phosphoribosyl 5 aminoimidazole 4-carboxylic acid; AICAR, 5-phosphoribosyl 4-(N-succinocarboxamide) 5-aminoimidazole; AICAR 5-phosphoribosyl 4-carboxamide 5-aminoimidazole; FAICR, 5-phosphoribosyl 4-carboxamide-5-formaminoimidazole; IMP, Inosinic acid; GMP, Guanylic acid; XMP, Xanthanylic acid; AMPS, Adenylosuccinate; AMP, Adenylic acid, Guo, Guanosine; G, Guanine; XO, Xanthosine; X, Xanthine; Ino, Inosine; Hx, Hypoxanthine; Ado, Adenosine; A, Adenine; R-1-P, Ribose-1-phosphate.

**Figure 2** The enzymes catalysing the numbered reactions are as follows:

| | | |
|---|---|---|
| 1 | Carbamoyl phosphate synthase | EC 2.1.3.2 |
| 2 | Aspartate transcarbamylase | EC 2.5.2.3 |
| 3 | Dihydroorotase | EC 6.3.4.16 |
| 4 | Dihydroorotate dehydrogenase | EC 1.3.3.1 |
| 5 | Orotate phosphoribosyl transferase | EC 2.4.2.10 |
| 6 | Orotate monophosphate decarboxylase | EC 4.1.1.23 |
| 7 | Thymidine phosphorylase | EC 2.4.2.4 |
| 8 | Thymidine kinase | EC 2.7.1.21 |
| 9 | Thymidylate synthase | EC 2.1.1.45 |
| 10 | Uridine kinase | EC 2.42.3 |
| 11 | Uridine phosphorylase | EC 2.4.2.3 |
| 12 | Uridylate kinase | EC 2.7.1.48 |
| 13 | Ribonucleotide reductase | EC 1.7.14.1 |
| 14 | Nucleotidase | EC 3.1.3.5 |
| 15 | Dihydrouracil dehydrogenase | EC 1.3.1.1 |

Abbreviations: $CO_2$, carbon dioxide; $NH_3$, ammonia; ATP, adenosine triphosphate; CP, carbamyl phosphate; C-L-A, Carbamyl L-aspartate; 4.5DHO, L-dihydroorotic acid; OR, orotic acid; OR5P, orotidine 5 phosphate; UMP, uridine 5 monophosphate; UDP, uridine diphosphate; dUDP, deoxyuridine diphosphate; dUMP, deoxyuridine monophosphate; UdR, uridine; U, Uracil; dTMP, deoxythymidine monophosphate; TdR, thymidine; T, thymine.

several enzymes of purine metabolism have been noted in both normal and tumour tissue. HPRT activity was elevated relative to normal tissue in the majority of tumour samples (breast and intestine) with levels ranging 0.2–2.0 U $mg^{-1}$. Nucleoside phosphorylase (NP) and ADA were also raised in tumour tissue whereas AP and 5NT activities were unchanged (Camici *et al.*, 1990). Although no attempt was made to correlate levels of expression with growth rate or proliferative fraction, the range of values suggests that they may be strongly influenced by some of the mechanisms discussed below.

In studies in this laboratory, in which four different human tumour lines (EJ bladder carcinoma, HOC8 ovarian carcinoma, MCF7 and HDA 231 breast carcinoma) were tested for methotrexate resistance under identical conditions a spectrum of resistance was observed which was unrelated to growth rate and phenotype (Kinsella unpublished observations).

No detailed molecular mechanism has been proposed to explain the altered balance of *de novo* and salvage synthesis observed by Weber *et al.* One possibility, suggested by Dutrillaux and Mulerio (1986), relates to the chromosome imbalance frequently observed in tumours. Using colorectal carcinoma as an example they have documented specific gains and losses involving a duplication of chromosome 17p which carries the thymidine kinase gene, a loss of chromosome 18 which carries the gene for thymidylate synthase and deletions in chromosome 5 which carries the gene for DHFR. Whilst such chromosome changes may make a contribution to altering the balance of *de novo* and salvage nucleotide synthesis, this is probably too simplistic an interpretation as there may be many compensatory changes in gene expression. Presently available data (Table I) indicate that there are genes coding for enzymes involved in nucleic acid metabolism on most human chromosomes and in some cases genes coding for enzymes catalysing successive steps are present in the same region of the chromosome e.g. 21q22.1 (Table I). This and other evidence quoted in Lai *et al.* (1991) suggests that genes in close physical proximity are co-ordinately regulated. The Ade$^-$D locus of Chinese hamster cells which encodes phosphoribosyl aminoimidazole carboxylase activity and maps to chromosome 4 together with the gene for phosphoribosyl pyrophosphate amidotransferase may be another example of the coalescence of related genetic information and co-ordinate regulation (Barton *et al.*, 1991). In *de novo* pyrimidine biosynthesis the first three enzymes also map in the same region of the chromosome 2p22.p21 and form the multifunctional CAD protein.

Other possible regulatory mechanisms include the loss or loss of function of purine responsive elements from the 5′ end of the HPRT gene (Walsh *et al.*, 1990) or of other regulatory elements such as those which occur in introns 1 and 2 of the human HPRT gene (Reid *et al.*, 1990). In addition studies of methylation patterns of many genes have shown that there is an inverse correlation between the level of methylation and gene activity (Feinburg & Volgstein, 1983; Doerfler, 1983; Riggs & Jones, 1983). In particular specific sites have been identified within and surrounding the mouse and human HPRT genes whose methylation status correlates well with HPRT activity. The region 400bp 3′ of exon 1 is extensively methylated in the inactive X chromosome of mouse and humans and completely unmethylated in the active X chromosome (Yen *et al.*, 1986). A second region of differential methylation has been identified in the 3′ 20 Kb of the gene which spans exons 3–9. The sites in this region are completely methylated on the active X and unmethylated on the inactive X (Lock *et al.*, 1986).

Methylation patterns can also be altered by drug exposure. Sites in the 5′ region of the hamster TK gene became unmethylated in MNNG induced revertants of TK$^-$ cells (Barr *et al.*, 1986). In addition structural rearrangements, either spontaneous or drug induced, may bring genes into proximity with new regulatory elements which act on gene promotors or may modify the binding of sequence specific regulatory elements by altering chromatin structure.

Support for the circumvention hypothesis comes from the work of Kinsella (1991) in studies of drug-resistance in human embryo fibroblasts of common genetic origin, but exhibiting phenotypes from normal to aggressively tumouri-

**Table I** Chromosomal location of genes involved in nucleic acid metabolism

| *Gene symbol* | *Protein* | *E.C. Number* | *Chromsome location* |
|---|---|---|---|
| AK2 | adenylate kinase 2 | 2.7.4.3 | 1p34 |
| AMPS | adenylosuccinate synthetase | 6.3.4.4 | 1cen-1q12 |
| AMPD1 | AMP deaminase 1 | 3.5.4.17 | 1p13-1p13 |
| AMPD2 | AMP deaminase 2 | 3.5.4.17 | 1p13-1p13 |
| UMPK | uridine monophosphate kinase | 2.7.1.48 | 1p32 |
| GUK1 | guanylate kinase 1 | 2.7.4.8 | 1q32-q42 |
| GUK2 | guanylate kinase 2 | 2.7.4.8. | 1q |
| CAD | carbamoylphosphate synthase | 2.1.3.2. | |
| | aspartate transcarbamylase | 2.5.2.3. | 2p22-p21 |
| | dihydroorotase | 6.3.4.16 | |
| ADACP2 | adenosine deaminase complexing protein 2 | | 2p23-qter |
| RRM2 | ribonucleotide reductase M2 polypeptide | 1.17.4.1 | 2p25-p24 |
| UMPS | uridine monophosphate synthetase (OPRT) | 2.4.2.10 | 3q13 |
| PPAT | phosphoribosyl pyrophosphate amidotransferase | 2.4.2.14 | 4pter-q21 |
| DHFR | dihydrofolate acid reductase | 1.5.1.3 | 5q11.2-q13.2 |
| ADACP1 | adenosine deaminase complexing protein 1 | | 6 |
| NT5 | 5′nucleotidase | 3.1.3.5 | 6q14-q21 |
| UP | uridine phosphorylase | 2.4.2.3 | 7 |
| PRPS1L1 | Phosphoribosylpyrophosphate synthetase 1-like 1 | | 7p22-qter |
| AK1 | adenylate kinase 1 | 2.7.4.3 | 9q34.1-q43.2 |
| AK3 | adenylate kinase 3 | 2.7.4.3 | 9p24-p13 |
| MTAP | methyl thioadenosine phosphorylase | 2.4.2.28 | 9pter-q12 |
| PRPS1L2 | Phosphoribosylpyrophosphate synthetase 1-like 2 | | 9 |
| ADK | adenosine kinase | 2.7.1.20 | 10cen-q24 |
| RRM1 | ribonucleotide reductase M1 polypeptide | 1.7.14.1 | 11p15.5-p15.4 |
| PFGS | phosphoribosylglycinamide formyltransferase | 2.1.2.2 | 14 |
| NP | nucleoside phophorylase | 3.4.2.1 | 14q13.1 |
| MTHFD | 5,10-methylenetetrahydrofolate dehydrogenase | 1.5.1.5 | |
| | 5,10-methylenetetrahydrofolate cyclohydrolase | 3.5.4.9 | 14q24 |
| | 10-formyltetrahydrofolate synthetase | 6.3.4.3 | |
| TK1 | thymidine kinase (mitochondrial) | 2.7.1.21 | 16 |
| APRT | adenine phosphoribosyl transferase | 2.4.2.7 | 16q24 |
| TK2 | thymidine kinase (soluble) | 2.7.1.21 | 16 |
| UMPH2 | UMP phosphohydrolase 2 | 3.3.3.5 | 17q23-q25 |
| TS | thymidylate synthase | 2.1.1.45 | 18pter-q12 |
| ADA | adenosine deaminase | 3.5.4.4 | 20q12-q13.11 |
| PAIS | phosphoribosylaminoimidazole synthetase | 6.3.3.1 | 21q22.1 |
| PRGS | phosphoribosylglycinamide synthetase | 6.3.4.13 | 21q22.1 |
| PGFT | phosphoribosylglycinamide formyltransferase | 2.1.2.2 | 21q22.1 |
| ADSL | adenylosuccinate lyase | 4.3.22 | 21q12-qter |
| PRPS1 | phosphoribosyl pyrophosphate synthetase 1 | 2.4.2.17 | Xq21-q27 |
| PRPS2 | phosphoribosyl pyrophosphate synthetase 2 | 2.4.2.17 | Xpter-q21 |
| HPRT | hypoxanthine phosphoribosyl transferase | 2.4.2.8 | Xq26 |
| MTHFDL1 | 5,10-methylenetetrahydrofolate dehydrogenase | | |
| | 5,10-methylenetetrahydrofolate cyclohydrolase | 3.5.4.9 | Xp11.3-p11.1 |
| | 10-formyltedrahydrofolate synthetase – like 1 | | |

Data from HMG 10.5, *Cytogenet. Cell Genet.*, Vol **55**, (1990).

genic Kinsella *et al.* (1990). Clonogenic measurements made in the presence of foetal bovine serum of the intrinsic sensitivities of these cells to the two cytotoxic drugs MTX and PALA showed that resistance to both drugs increased with the progression to morphological transformation, anchorage independence and growth in nude mice. The difference in resistance between the immortal and tumourigenic cell lines was eliminated for both drugs when the experiments were repeated in dialysed foetal bovine serum, but could be restored by the addition of hypoxanthine in the case of resistance to MTX and by the addition of uridine in the case of resistance to PALA. No evidence for the presence of amplified DHFR or CAD genes was found in any of these cell lines which was consistent with their lack of stable resistance. These observations suggested an important role for the salvage pathways of purine and pyrimidine biosynthesis in the increased resistance of the more tumourigenic cell lines.

PALA is a powerful inhibitor of *de novo* pyrimidine biosynthesis whilst MTX, acting on DHFR, inhibits both purine and pyrimidine biosynthesis. Resistance to both drugs is commonly but not always (Kinsella & Fox, 1988) due to amplification, which in the case of resistance to MTX is DHFR (Schimke *et al.*, 1977) and in the case of resistance to PALA, the multi-functional CAD gene (Wahl *et al.*, 1979; Zieg *et al.*, 1983; Meinkoth *et al.*, 1987). The relationship between gene amplification and tumourigenicity, as assessed by resistance to MTX and PALA has recently been studied in mouse fibrosarcoma (Cillo *et al.*, 1989) and in rat liver cell lines (Otto *et al.*, 1989) exhibiting different degrees of tumourigenicity. In both studies a striking parallel was observed between the acquisition of drug resistance due to gene amplification and increasing tumourigenicity. However, although gene amplification is usually responsible for the high frequency of resistance to these drugs following *in vitro* selection, amplification of genes mediating drug resistance has been reported in very few tumours (Wright *et al.*, 1990 and refs therein). The ability of cells to salvage nucleosides may facilitate amplification by allowing them to survive long enough for re-replication to occur. However, this is not the mechanism operating in the study of Kinsella (1991).

These data, although preliminary, support the concept of a progression-linked change in the key metabolic pathways of purine and pyrimidine synthesis (Weber, 1983). Not surprisingly, inhibitors of *de novo* pyrimidine synthesis, such as PALA, produce a reduction in the intracellular pyrimidine ribonucleoside (UTP and CTP) and deoxyribonucleoside (dCTP) triphosphate pools (Plagemann & Behrens, 1976; Jayaram *et al.*, 1979; Moyer & Handschumacher, 1979; Moyer *et al.*, 1981; Low & Kufe, 1981). Moreover, the antiproliferative, cytotoxic and antitumour effects of PALA can be reversed by the addition of exogenous uridine (Johnson, 1977; Cadman & Benz, 1980; Karle *et al.*, 1980; 1984) and partially by deoxycytidine (Bhalla & Grant, 1987). What all these exogenous agents have in common is that they are either substrates of the salvage pathway enzymes or components of the *de novo* pathway distal to the inhibitory block.

### *Plasma levels of nucleic acid components*

If the circumvention hypothesis is to have any validity for the clinical situation then we must ask whether purines and pyrimidines are present in the plasma in sufficient concentrations to compete with achievable plasma concentrations of drug analogues. Typical of the base analogues used in therapy is 6MP for which plasma concentrations of 10 μM and ~0.1 μM were achieved immediately after oral and intravenous administration respectively, but these levels declined rapidly over the subsequent 6 h (Zimm *et al.*, 1984). Intravenous infusion over 48 h resulted in the maintenance of a mean plasma concentration of 6.9 μM without host toxicity (Zimm *et al.*, 1985). In comparison, median values (μM) of adenosine, inosine and hypoxanthine in plasma from seven normal individuals at rest were 0.2, 0.6 and 1.3 respectively (Sinkeler *et al.*, 1986). Plasma hypoxanthine levels were reported for 16 normal subjects (range 0.2–1.9 μM, mean 0.56 μM), ten untreated leukaemia patients (0.1–1.1 μM, mean 0.68 μM) and 14 solid tumour patients (0.3–2.6 μM, mean 0.89 μM) (Wong & Howell, 1984). Thus plasma hypoxanthine levels varied over a 10-fold range in both normal and cancer bearing individuals. Plasma adenosine levels in venous and arterial blood were reported at 0.15 ± 0.03 μM ($n = 15$) respectively (Solleri *et al.*, 1987).

Even higher concentrations of nucleotides are present in human plasma. It is generally accepted that concentrations of adenine nucleotides are about 20–30 μM (Gordon, 1985). Other determinations of adenine nucleotides (ATP + ADP + AMP) using HPLC have indicated concentrations ranging from 2–35 μM (Brankiewicz, personal communication).

High local concentrations of nucleotides and nucleosides will occur as a result of destruction of tumour cells by cytotoxic therapy. In addition, extracellular ATP concentrations of > 50 μM make erythrocytes semipermeable so that they release more ATP from their cytoplasm. ATP is also released from erythrocytes and platelets as a result of traumatic shock and during inflammatory reactions, at concentrations ranging between 200 μM and 1 mM (Gordon, 1985).

### *Ecto-enzyme cascade generates nucleosides*

Nucleosides can be derived from these sources by extracellular nucleotidases present in serum and by ectoenzymes bound to the external face of the plasma membrane (Figure 3). Thus ecto-ATPase, ecto-ADPase, ecto-ADP kinase and 5′ nucleotidase, present on a variety of cell types (Boyle *et al.*, 1989; Gutensohn & Rieger, 1986), enable the conversion of nucleosidetriphosphates (principally ATP, but also GTP, UTP and CTP at lower rates) to their respective nucleosides. Plasma nucleotide concentrations will, therefore, represent a balance between release of nucleotides and their degradation by extracellular nucleotidases. Thus is would appear that sufficient concentrations of nucleosides are likely to be available *in vivo* to produce significant rescuing effects during drug-induced cytotoxicity.

### *Nucleoside transporters control influx and efflux*

The uptake of nucleosides resulting from ectoenzyme activities is mediated by nucleoside transporters (Cass *et al.*, 1987). Non-concentrative, facilitated diffusion of a broad range of purine and pyrimidine nucleosides is controlled by kinetically symmetrical transporters which fall in two classes with respect to their sensitivity to inhibition by NBMPR, dilazep and dipyridamole. Sensitive transporters possess high affinity sites for binding NBMPR which inhibits their activity in nanomolar amounts. Resistant transporters lack the high affinity site and are only inhibited by NBMPR at concentrations above 1–10 micromolar. These also possess a broad specificity but their affinity for some nucleosides may differ from that of the sensitive transporters. The properties of the transporter proteins have been reviewed (Plagemann *et al.*, 1988).

Many cells, including the Morris 3924A rat hepatoma used in several of the studies by Weber and coworkers, express both forms of transporter and there is genetic evidence from mouse S49 (Cohen *et al.*, 1985; Aronow *et al.*, 1985) and L1210 cells (Belt & Noel, 1988) that the two forms may be coded by different genes. Other cells appear to express only one type of transporter, e.g. human erythrocytes and mouse S49 lymphoma cells are $NBMPR^s$ whereas Walker 256 sarcoma and Novikoff hepatoma cells are $NBMPR^r$ (see review by Paterson *et al.*, 1987).

Active transport of nucleosides has been observed in epithelial cells of rat (Jakobs & Paterson, 1986) and rabbit intestine (Jarvis, 1989*a*) and rat kidney (Le Hir & Dubrach, 1985), murine splenocytes (Plagemann & Woffendin, 1989) and guinea pig enterocytes (Schwenk *et al.*, 1984). This system is concentrative and stimulated by sodium ions, although a component of the rat kidney transporter is also stimulated by potassium ions. The $K_m$ of this type of trans-

**Figure 3** Schematic representation of extracellular purine nucleotide metabolism and the pentose phosphate shunt. Also shown are purine nucleoside and other transporters known to be involved in drug uptake. es, equilibrative nucleoside transporter sensitive to NBMPR; ei, equilibrative nucleoside transporter insensitive to NBMPR; cif, $Na^+$ dependent concentrative transporter; gp170, efflux transporter encoded by *mdr* gene or genes; ct, choline transporter utilised by nitrogen mustard ($HN_2$); aatp, amino acid transporter proteins utilised by melphalan.
→ indicates drug uptake by passive diffusion. Thick arrows at es and ei indicate that efflux is more important than influx: Antimetabolite drugs utilise ei, es and cif to varying extents in different cell lines. Enzymes are in lower case letters; substrates in upper case. All other abbreviations are specified elsewhere in the text.

porter is about 20-fold lower than that of facilitated diffusion transporters, hence at low nucleoside concentrations active transport predominates whilst at higher concentrations both types of transporter are active. Sodium-dependent transporters are resistant to NBMPR, dilazep and dipyridamole. In addition to nucleoside transporters, evidence is accumulating for the occurrence of high affinity nucleobase transporters (see for example, Beck & Ullman, 1989). A recent overall review of transport systems was provided by Jarvis (1989*b*).

Because different tissues vary in the types of nucleoside transporter they express, the cytotoxicity of nucleoside drugs can be abrogated to a greater or lesser extent by NBMPR, dilazep and dipyridamole, a feature that has suggested the possibility of selective protection of normal tissues by inhibitors. Thus Kaplinsky *et al.* (1986) found that NBMPR protected normal tissues but not $NBMPR^r$ neuroblastoma cells against tubericidin (7-deazaadenosine). A similar strategy has been suggested for protecting highly sensitive bone marrow cells when using tubericidin to treat inhibitor resistant leukaemia (Cass, 1989).

The finding that the cytotoxicity of some nucleoside drugs can be reduced by transport inhibitors while that of others is unaffected indicates that different drugs may use various transporters that have different nucleoside specificities. Prus *et al.* (1990) found that transport inhibitors reduced the cytotoxicity of tubericidin to MOLT 4 and CCRF CEM T cell lymphoblastic leukaemia cell lines but had no effect on the cytotoxicity of 9-β-D-arabino-furanosylguanine (araG) on the cells.

*A three component pathway mediates reutilisation of nucleotides*

The salvage of nucleosides and nucleobases can thus be viewed as a three component system comprising the ecto-enzyme cascade, carrier mediated transport across the plasma membrane followed by intracellular phosphorylation or phosphoribosylation by kinases, e.g. thymidine kinase (TK) or by phosphoribosyltransferases specific for hypoxanthine and guanine (HPRT) and adenine (APRT).

The kinetic properties of these components are such as to generate very effective salvage systems. Extracellular nucleotidases are especially active in endothelial cells, smooth muscle cells, B lymphoblasts and platelets. The $K_m$ value for pig aorta endothelial cell 5NT was 28 μm (Gordon, 1985) and the apparent $K_m$ for human fibroblasts was 38 nM per $10^6$ cells (Boyle *et al.*, 1989). Apparent $K_m$ values of the lymphoblastoid cell line BHG-83-1 for ATPase and ADPase were 20 μM and 50 μM respectively (Gutensohn & Rieger, 1986).

The $K_m$ values of nucleoside transporters for a number of drug analogues are known to be of the same order of magnitude as those for their normal substrates, e.g. adenosine 50–150 μM, tubericidin 50–120 μM; thymidine 150–250 μM, 5-iodo-2-deoxyuridine 90 μM. Both 6-thioguanine and 6-mercaptopurine are efficiently transported by the hypoxanthine carrier ($K_m$ 200–400 μM) whereas 8-azaguanine differs in that it diffuses through the membrane in its non-ionised form ($pK_a$ 6.6) (Plagemann *et al.*, 1981). 5-Flourouracil is as efficiently transported by the uracil carrier as is uracil ($K_m$ 15 mM) but again only in its non-ionised form ($pK_a$ 8.0) (Wohlhueter *et al.*, 1980).

Phosphorylation or phosphoribosylation involves high affinity reactions with $K_m$ values between 1–100 μM. The $K_m$ values of HPRT for the natural substrates hypoxanthine and guanine are similar to those for the cytotoxic analogues 6TG and 6MP (Table II). Some variation is seen between values for different cell types and between species. Only one study however (Kong & Parks, 1974) was on purified enzyme, where the $K_m$ was shown to depend on pH.

Several additional lines of evidence indicate the biological importance of the ecto-enzyme/nucleoside transporter/intracellular phosphorylation route for purine and pyrimidine salvage and by-pass of drug cytotoxicity.

(1) IMP prevented cytotoxicity caused by MTX in B lymphoblastoid cells only if they expressed 5′ nucleotidase (5NT) (Thompson, 1986).
(2) The growth inhibitory effects of high concentrations (> 50 μM) of ATP, ADP, AMP and adenosine on mouse 3T6 cells were prevented by inhibitors of adenosine transport, dipyridamole and NBMPR (Weisman *et al.*, 1988).
(3) Inhibition of *de novo* purine and pyrimidine synthesis by acivicin caused cytotoxicity which was synergistically enhanced by dipyridamole (Weber, 1983; Fisher *et al.*, 1984).

*Synergistic effects of dipyridamole suggest salvage pathway involvement*

In cancer therapy the mechanism of action of dipyridamole (DPM, persantin) is primarily through inhibition of nucleoside transporters, however in interpreting its synergistic effects on cancer chemotherapy other mechanisms may also operate and must be born in mind (Figure 3). Since its introduction in 1959 DPM has become widely used in the treatment of cardiovascular disease because it prevents platelet aggregation, has vasodilatory activity and is non-toxic (Fitzgerald, 1987). The mechanism of inhibition of platelet aggregation was thought to involve inhibition of platelet cyclic AMP phosphodiesterase, however this effect is rather weak and recently the elevation of plasma adenosine by blockage of erythrocyte nucleoside transporters has been proposed (Luthje, 1989). The adenosine then binds to specific receptors on the platelet surface, causing activation of adenylate cyclase and elevation of cyclic AMP levels associated with inhibition of platelet function. There are also reports that DPM alters plasma membrane properties (Sowemimo-Coker *et al.*, 1983; Verscheuruen *et al.*, 1983), potentiates the inhibition of virus replication (Szebeni *et al.*, 1989) and induces interferon (Galabov & Mastikova, 1982). Whilst it is possible that some of these effects are secondary to inhibition of nucleoside transport, there are clear indications from the above examples that DPM also affects other cellular pro-

**Table II** Km values for various substrates (μM)

| *Enzyme* | *Cell type* | *Hypoxanthine* | *Guanine* | *Adenine* | *6TG* | *6MP* | *Reference* |
|---|---|---|---|---|---|---|---|
| HPRT | Mouse sarcoma | | 5.4 | | 4.0 | | Van Diggelen *et al.*, (1979) |
| | Chinese hamster V79A | 10.0 | – | | 3.0 | | – |
| | V79S | 10.0 | – | | 12.0 | | Fox & Hodgkiss (1981) |
| | Human erythrocytes | | 5.2 | | 12.8 | 14.0 | Kong & Parks (1974) |
| | pH7.0 | | | | | | McDonald & Kelly (1971) |
| | pH7.0 | 5.0 | 17.0 | | | | |
| | Human lymphoblasts | 74.0 | | | | | |
| APRT | Human lymphoblasts | | | 33.0 | | | Wood *et al.* (1973) |

cesses. For this reason potentiation by DPM (and probably NBMPR and dilazap also) should only be taken as a first indication of the involvement of nucleoside transport in a process and should be supported by biochemical data.

Since DPM inhibits kinetically symmetrical non-concentrative nucleoside transporters as well as the sodium-dependent concentrative transporters, it may be expected to inhibit both influx and efflux of nucleosides. Sometimes these functions appear to be selectively used by different molecules. Thus DPM prevents repletion of intracellular nucleotide pools by blocking influx of normal nucleosides and can also prevent efflux of fluorodeoxyuridine which leads to elevation of fluorodeoxyuridine monophosphate and the consequent inhibition of thymidylate synthase as a means of enhancing the cytotoxicity of 5FU (Grem & Fisher, 1985; Alberts *et al.*, 1987). This rationale formed the basis of phase I trial of DPM with 5FU and folinic acid (Budd *et al.*, 1990). A confounding factor with such treatments *in vivo* is the presence in blood of proteins, principally $\alpha_1$-acid glycoprotein, which bind most of the DPM. The variable concentration of $\alpha_1$-acid glycoprotein in different individuals may represent a pharmacogenetic component of drug resistance (Piafsy & Borga, 1977).

Thymidylate synthase is also inhibited by the quinazolene antifolate, CB3717, which caused growth inhibition of A549 human lung carcinoma cells that could be overcome by salvage of exogenous thymidine (Curtin & Harris, 1988). The cytotoxicity of CB3717 was increased by the presence of DPM or by the use of dialysed serum to reduce the availability of exogenous thymidine. DPM was shown to inhibit influx of TdR by >95% and to inhibit efflux of TdR by 61% and UdR by 89%. The authors argued that these effects would exacerbate the nucleotide pool imbalance caused by inhibition of thymidylate synthase and so contribute to cytotoxicity.

Although we have emphasised the effects of DPM on nucleoside transport there are also indications that transport of other molecules may be inhibited (Kessel & Dodd, 1972). DPM inhibited the uptake of thymidine by sarcoma 180 cells but also inhibited the efflux of methotrexate (Cabral *et al.*, 1984; Nelson & Drake, 1984). Sarcoma 180 was also used with Hela cells to investigate the potentiation of adriamycin toxicity by DPM (Kusumoto *et al.*, 1988). DPM caused a 2.4-fold decrease in the $LD_{50}$ of adriamycin with 1.4-fold increase in drug uptake. Using human ovarian carcinoma 2008 cells Howell and coworkers demonstrated synergism between DPM and cisplatin (Howell *et al.*, 1987), etoposide (VP-16) (Howell *et al.*, 1989*a*,*b*) doxorubicin and vinblastine (Howell *et al.*, 1989*b*). In each case DPM increased the steady state concentrations of the drugs. Only with vinblastine was this accompanied by an increase in the initial influx: with all drugs the initial rate of efflux was inhibited but not sufficiently to account for the increased steady state concentration. On this basis it was suggested that DPM was also affecting other, undefined, mechanisms controlling drug concentrations. The possibility that DPM was inhibiting efflux mediated by the gp170 MDR1 gene product was discarded because 2008 cells are relatively sensitive to the drugs used and hence it was thought unlikely that there would be sufficient gp170 present for DPM to cause inhibition of efflux at the magnitude observed. Interaction of DPM with gp170 needs to be specifically addressed in sensitive and resistant isogenic cell lines.

There are also numerous other examples, Grem and Fisher (1985), Nelson and Drake (1984), Fisher *et al.* (1984), Cabral *et al.* (1984), Sobreso *et al.* (1985) and Chan *et al.* (1989), of the sensitisation of cells to the cytotoxic effects of MTX, PALA and fluorouridine by nucleoside transport inhibitors and of the rescue from 5-fluorouracil toxicity by uridine.

### *Resistance to 6-mercaptopurine is multifactorial*

The ecto- and cytosolic forms of NT, may be involved in drug resistance in different ways. The ecto form participates in the ecto-enzyme/nucleoside transporter system that recycles normal nucleotides which compete with toxic analogues, whereas the cytosolic enzyme may participate in the dephosphorylation of toxic nucleotides. These alternatives were offered as explanations (Pieters & Veerman, 1988) of the observation that children with common-ALL showed a higher probability of complete remission if the leukaemic cells were $NT^-$ than if they were $NT^+$ (Veerman *et al.*, 1985). The maintenance therapy involved the use of 6-mercaptopurine and MTX.

Levels of thioguanine nucleotides (TGN), the major cytotoxic metabolites of 6MP varied 6.6-fold when measured in erythrocytes of 120 children with ALL (Lennard & Lilleyman, 1989). Treatment success was correlated with the attainment of high TGN levels, hence factors such as catabolic pathways that reduce TGN levels will contribute to poorer survival. Catabolism occurs via thiopurine methyltransferase (TPMT) to 6-methyl mercaptopurine, via xanthine oxidase to 6-thiouric acid (Lennard & Lilleyman, 1987) and potentially through dephosphorylation mediated by cytosolic NT (Pieters *et al.*, 1987).

As described by Lennard and Lilleyman (1989), 6MP is subject to first pass detoxification through the intestine and liver by xanthine oxidase (XO). Although inter-individual variation in expression of this enzyme in tissues is not significant, there may be considerable variation in the amount of XO consumed in food as a consequence of differences in diet. On the other hand, TPMT shows monogenic inheritance of two co-dominantly expressed alleles, $TPMT^L$ and $TPMT^H$, controlling low and high levels of activity in erythrocytes and lymphocytes (Weinshilboum & Sladek, 1980; Van Loon & Weinshilboum, 1982). Individuals homozygous for L/L occur at a frequency of 1 in 300 and 10% of the population is heterozygous (L/H).

Variations in NT activity are found within blood lymphocytes where expression is associated with differentiation and in leukaemias which appear to reflect blockages in differentiation. Thus 'differentiation-arrested' leukaemias such as T acute lymphoblastic leukaemia have low NT activity, whereas 'end cell' leukaemias such as common acute lymphoblastic leukaemia have high NT activity (Gutensohn *et al.*, 1984). However there is a caveat that these correlations have generally been made by assaying ecto- rather than cytosolic-NT activity. Although Boyle *et al.* (1989) could find no evidence for separate genes encoding the two forms of NT in fibroblasts they only assayed for the 'low $K_m$' activity described by Spychala *et al.* (1988). This appears to be a ubiquitous activity which has a preference for AMP and pyrimidine nucleoside monophosphates with micromolar $K_m$ values and is inhibited by ATP. In contrast a 'high $K_m$' activity prefers IMP and GMP with millimolar $K_m$ values, is stimulated by ATP other nucleoside triphosphates and glycerate 2,3-biphosphate (Bontemps et al., 1989) and is inhibited by $P_i$. Thus the 'low $K_m$' enzyme has properties similar to those of ecto-NT but those of the 'high $K_m$' enzyme appear to be distinct. Among other tissues the 'high $K_m$' enzyme is found in human lymphocytes, but it is not known yet which of the soluble activities show a preference for TGN or how they are expressed during lymphocyte differentiation.

A further major determinant of the response to MTX and 6MP therapy is the availability of PRPP. Methotrexate causes inhibition of *de novo* synthesis and results in increased PRPP availability. The increased PRPP levels can then be used for enhanced incorporation of 6MP. Molt 4 (T) Raji (B) and KM3 (non T non B) human lymphoblastoid cells have been compared with respect to the activities of purine *de novo* (PDNS) and salvage pathways. Molt 4 showed high activity of both pathways, Raji had low PDNS and an active salvage whereas both pathways were moderately active in KM3. The time course of elevation of PRPP levels was measured after MTX exposure (0.02 μM and 0.2 μM) in all three lines and the amount of hypoxanthine and 6MP incorporated was shown to be directly correlated at each time point with the PRPP level. The absolute amount of 6MP incorporated correlated with the activity of the purine salvage pathway. Overall the data indicate that Raji and Molt 4 cells will be more sensitive to the cytotoxic effect of MTX plus 6MP than KM3 cells (Bokkerink et al., 1988*a*,*b*).

### *Perspectives*

In this review we have cited evidence in support of the hypothesis that salvage of nucleotides involving an ecto-enzyme cascade, nucleoside transport and endogenous phosphorylation is a significant mechanism in resistance to a whole variety of antitumour agents. The underlying mechanism is the circumvention of the inhibition of key enzymes of *de novo* purine or pyrimidine synthesis as proposed by Natsumeda et al. (1989) on biochemical grounds. Our own quoted data (Kinsella, 1991) provide biological support for the idea and in this review we have emphasised the contribution of ecto-enzymes to the process. There is ample evidence that nucleosides are present in serum at concentrations sufficient to fulfill a salvage function and may be augmented locally from the nucleic acids of dying cells in a tumour. The ecto-enzymes involved in the nucleotide cascade are highly active at the observed physiological concentrations of nucleotides. As the genes involved in salvage and *de novo* synthesis are cloned into expression vectors, their transfection into non-expressing recipient cells offers a powerful means of testing the details and implications of the hypothesis. Salvage pathways appear to be particularly important in leukocytes, where deficiencies of adenosine deaminase, purine nucleoside phosphorylase and 5′ nucleotidase appear to be associated with suppression of immunological function. Similar deficiencies occur in some leukaemias and their investigation and correction by genetic engineering promises to illuminate our understanding of the role of salvage not only in drug resistance but also in the wider aspects of leucocyte differentiation and leukaemogenesis.

This work was supported by Grants from the Cancer Research Campaign.

### References

ALBERTS, D.S., EINSPAHR, J., PENG, Y.M. & SPEARTS, P. (1987). Dipyridamole potentiation of FUdR antitumour activity. *Clin. Pharmacol Therapeut.*, **41**, 247.

ARONOW, B., ALLEN, K., PATRICK, J. & ULLMAN, B. (1985). Altered nucleoside transporters in mammalian cells selected for resistance to the physiological effects of inhibitors of nucleoside transport. *J. Biol. Chem.*, **260**, 6226.

BARR, F.G., RAJAGOPALAN, S., MACARTHER, C. & LIEBERMAN, M. (1986). Genomic hypomethylation and far 5′ sequence alterations are associated with carcinogen induced activation of the Hamster TK gene. *Mol. Cell Biol.*, **6**, 3032.

BARTON, J.W., HART, I.M. & PATTERSON, D. (1991). Mapping of a locus correcting lack of phosphoribosylamino imidazole carboxylase activity in Chinese hamster ovary cell Ade$^-$ D mutants to chromosome 4. *Genomics*, **9**, 314.

BECK, J.A. & ULLMAN, B. (1989). Expression of a novel high affinity purine base transport system in mutant mouse S49 cells does not require a functional nucleoside transporter. *Adv. Exp. Med. Biol.*, **253B**, 525.

BELT, J.A. & NOEL, L.D. (1988). Isolation and characterisation of a mutant of L1210 murine leukemia deficient in nitrobenzylthioinosine insensitive nucleoside transport. *J. Biol. Chem.*, **263**, 13819.

BHALLA, K. & GRANT, S. (1987). Effect of deoxycytidine on the *in vitro* response of human leukaemia cells to inhibitors of *de novo* pyrimidine biosynthesis. *Cancer Chemother. Pharmacol.*, **19**, 226.

BOKKERINK, J.P.M., BAKKER, M.A.H., HULSCHER, T.W., DEABREU, R.A. & SCHRETLAN, E.D.A.M. (1988*a*). Purine *de novo* synthesis as the basis of synergism of methotrexate and 6 mercaptopurine in human malignant lymphoblasts of different lineages. *Biochem. Pharmacol.*, **37**, 2321.

BOKKERINK, J.P.M., DEABREU, R.A., BAKKER, M.A.H. & 4 others (1988*b*) Effects of methotrexate on purine and pyrimidine metabolism and cell kinetic parameters in human malignant lymphoblasts of different lineages. *Biochem. Pharmacol.*, **37**, 2329.

BONTEMPS, F., VINCENT, M.F., VAN DEN BERGH, F., VAN WAEG, G. & VAN DEN BERGH, G. (1989). Stimulation by glycerate 2,3-bisphosphate: a common property of cytosolic IMP-GMP 5′-nucleotidase in rat and human tissues. *Biochim. Biophys. Acta*, **997**, 131.

BOYLE, J.M., HEY, Y. & FOX, M. (1989). Nucleotide ectoenzyme activities of human and Chinese hamster fibroblasts in tissue culture. *Biochem. Genet.*, **27**, 655.

BRENNAND, J. & CASKEY, C.T. (1985). The molecular basis of genetically acquired resistance to purine analogues in cultured mammalian cells. In *Antitumour Drug Resistance*, Fox, B.W. & Fox, M. (eds), pp. 88–99, Springer-Verlag: Berlin, Heidelburg, New York, London.

BRONSTEIN, A., LELEIKO, N.S. & MUNRO, H.N. (1983). RNA synthesis by villus and crypt cell nuclei of rat epithelium. *Biochim. Biophys. Acta*, **739**, 334.

BUDD, G.T., JAYARAJ, A., GRABOWSKI, D. & 6 others (1990). Phase I trial of dipyridamole with 5-fluorouracil and folinic acid. *Cancer Res.*, **50**, 7206.

CABRAL, S., LEIS, S., BORER, L., NEMBOOT, M. & MOIDAN, J. (1984). Dipyridamole inhibits reversion by thymidine of methotrexate effect and increases drug uptake in Sarcoma 180 cells. *Proc. Natl Acad. Sci. USA*, **81**, 3200.

CADMAN, E. & BENZ, C. (1980). Uridine and cytidine metabolism following inhibition of *de novo* pyrimidine synthesis by pyazofurin. *Biochim. Biophys. Acta*, **609**, 372.

CAMICI, M., TOZZI, M.G., ALLEGRINI, S. & 5 others (1990). Purine salvage activities in normal and neoplastic human tissues. *Cancer Biochem. Biophys.*, **11**, 201.

CASS, C.E., BELT, J.A. & PATERSON, A.R.P. (1987). Adenosine transport in cultured cells and erythrocytes. In *Cardiac Electrophysiology and Pharmacology of Adenosine and ATP: Basic and Clinical Aspects*, Peleg, A., Michelson, E.L. & Dreifus, L.S. (eds), pp. 13–40, Alan R. Liss Inc: New York.

CASS, C.E. (1989). Modulation of activity of cytotoxic nucleosides against hematopoietic cells by inhibitors of nucleoside transport. *Cancer Chemoth. Pharmacol.*, **24**, A79.

CHAN, T.C.K. & JANOTA (1989). The role of membrane nucleoside transporter in natural and acquired drug resistance. *Cancer Chemother. Pharmacol.*, **24**, A78.

CILLO, C., LING, V. & HILL, R.P. (1989). Drug resistance in KHT fibrosarcoma cell lines with different metastatic ability. *Int. J. Cancer*, **43**, 107.

COHEN, A., LEUNG, C. & THOMPSON, E. (1985). Characterisation of mouse lymphoma cells with altered nucleoside transport. *J. Cell Physiol.*, **123**, 431.

CURTIN, N.J. & HARRIS, A.L. (1988). Potentiation of quinazoline antifolate (CB3717) toxicity by dipyridamole in human lung carcinoma, A549, cells. *Biochem. Pharmacol.*, **37**, 2113.

DEUCHARS, K.L. & LING, V. (1989). P-glycoprotein and multi-drug resistance in cancer chemotherapy. *Seminars in Oncol.*, **16**, 156.

DOERFLER, W. (1983). DNA methylation and gene activity. *Ann. Rev. Biochem.*, **52**, 93.

DUTRILLAUX, B. & MULERIO, M. (1986). Induction of increased salvage pathways of nucleotide synthesis by dosage effect due to chromosomal imbalances may be fundamental in carcinogenesis: the example of colorectal carcinoma. *Ann. Genet.*, **29**, 11.

FEINBURG, A.P. & VOLGSTEIN, B. (1983). Hypomethylation distinguishes genes of some human cancers from their normal counterparts. *Nature*, **301**, 89.

FISHER, P.H., PANCKI, R., BITTNER, G. & WILSON, J.K.V. (1984). Enhancement of the sensitivity of human colon cancer cells to growth inhibition by acivicin achieved by inhibition of nucleic acid precursor salvage by dipyridamole. *Cancer Res.*, **44**, 3355.

FITZGERALD, G.A. (1987). Dipyridamole. *New Engl. J. Med.*, **316**, 1247.

FOX, M. & HODGKISS, R.J. (1981). Mechanism of cytotoxic action of azaguanine and thioguanine in wild type V79 cell lines and their relative efficiency in selection of structural gene mutants. *Mutation Res.*, **80**, 165.

FOX, M. & ROBERTS, J.J. (1987). Drug resistance and DNA repair. *Cancer & Metastasis Rev.*, **6**, 261.

GALABOV, A.S. & MASTIKOVA, M. (1982). Dipyridamole is an interferon inducer. *Acta Virol.*, **26**, 137.

GOLDENBURG, G.J. & BEGLEITER, A. (1984). Alterations in drug transport. In *Antitumour Drug Resistance*. Fox, B.W. & Fox, M. (eds), pp 241–298. Springer Verlag: Berlin, Heidelburg, New York, Tokyo.

GORDON, J.L. (1985). Extracellular ATP, effects, sources and fate. *Biochem. J.*, **233**, 309.

GREM, J.L. & FISHER, P.H. (1985). Augmentation of 5-florouracil cytotoxicity in human colon cancer cells by dipyridamole. *Cancer Res.*, **45**, 2967.

GUTENSOHN, W. & RIEGER, J. (1986). Ectoenzymes of nucleotide metabolism on human lymphoid cells. *Adv. Exptl. Med. Biol.*, **195B**, 459.

GUTENSOHN, W., THEIL, E. & BUSCHETTI, S. (1984). Ecto-5' nucleotidase as a leukemia marker. *Adv. Exp. Med. Biol.*, **165B**, 249.

HOWELL, S.B., VICK, J. & ANDREWS, P.A. (1987). Biochemical modulation of cisplatin by dipyridamole. *Proc. AACR*, **28**, 313.

HOWELL, S.B., HOM, D.K., SANGA, R., VICK, J.S. & CHAN, T.C.K. (1989*a*). Dipyridamole enhancement of etoposide sensitivity. *Cancer Res.*, **49**, 4147.

HOWELL, S.B., HOM, D.K., SANGA, R., VICK, J.S. & ABRAHAMSON, I.S. (1989*b*). Comparison of the synergistic potentiation of etoposide, doxorubicin and vinblastine cytotoxicity by dipyridamole. *Cancer Res.*, **49**, 3178.

JAKOBS, E.S. & PATERSON, A.R.P. (1986). Sodium-dependent, concentrative nucleoside transport in cultured intestinal epithelial cells. *Biochem. Biophys. Res. Commun.*, **140**, 1028.

JARVIS, S.M. (1989*a*). Characterisation of sodium-dependent nucleoside transport in rabbit intestinal brush-border membrane vesicles. *Biochim. Biophys. Acta*, **979**, 132.

JARVIS, S.M. (1989*b*). Uniport carriers for metabolites. *Curr. Opin. Cell Biol.*, **1**, 721.

JAYARAM, H.N., COONEY, D.A., VISTICA, D.T., KARIYA, S. & JOHNSON, R.K. (1979). Mechanism of sensitivity or resistance of murine tumours to N-(phosphonoacetyl)-L-aspartate. *Cancer Treat. Rep.*, **63**, 1291.

JOHNSON, R.K. (1977). Reversal of toxicity and antitumour activity of N-(phosphonoacetyl)-L-aspartate by uridine or carbamyl-DL-aspartate *in vivo*. *Biochem. Pharmacol.*, **26**, 81.

KAPLINSKY, C., YEGER, H., ESTROV, Z. & 4 others (1986). Selective protection of tubercidin toxicity by nitrobenzyl thioinosine in normal tissues but not human neuroblastoma cells. *Cancer Chemother. Pharmacol.*, **17**, 264.

KARLE, J.M., ANDERSON, L.W., ERLICHMAN, C. & CYSYK, R.L. (1980). Serum uridine levels in patients receiving N-(phosphonoacetyl)-L-aspartate. *Cancer Res.*, **40**, 2938.

KARLE, J.M., ANDERSON, L.W. & CYSYK, R.L. (1984). Effect of plasma concentrations of uridine on pyrimidine biosynthesis in cultured L1210 cells. *J. Biol. Chem.*, **259**, 67.

KESSEL, D. & DODD, D.C. (1972). Effects of persantin on several transport systems of murine leukemias. *Biochim. Biophys., Acta.*, **288**, 190.

KINSELLA, A.R. (1991). Decreasing sensitivity to cytotoxic agents parallels increasing tumourigenicity in human fibroblasts. *Cancer Res.*, **51**, 1855.

KINSELLA, A.R., FIZER-MALIZEWSKA, F., MITCHELL, E.D., GUO, Y.P., FOX, M. & SCOTT, D. (1990). The introduction of the activated N-ras oncogene into human fibroblasts by retroviral vector induces morphological transformation and tumourigenicity. *Carcinogenesis*, **11**, 1803.

KINSELLA, A.R. & FOX, M. (1988). Phenotypic resistance to methotrexate and N-phosphonoacetyl L aspartate is induced by treatment with 12-0 tetradecanoylphorbol 13-acetate (TPA). *Int. J. Cancer*, **42**, 87.

KOHN, K.W., POMMIER, Y., KERRIGAN, D., MARKOVITS, J. & COVEY, J.M. (1987). Topoisomerase II as a target of anticancer drug action in mammalian cells. *Natl Cancer Inst. Monograph*, **4**, 61.

KONG, C. & PARKS, R.E. (1974). Human erythrocyte hypoxanthine guanine phosphoribosyl transferase: effect of pH on the enzymatic reaction. *Mol. Pharmacol.*, **10**, 648.

KUSUMOTO, H., MAEHARA, Y., ANAI, H., KUSUMOTO, T. & SUGIMACHI, K. (1988). Potentiation of adriamycin toxicity by dipyridamole against Hela cells *in vitro* and sarcoma 180 cells *in vivo*. *Cancer Res.*, **48**, 1208.

LAI, L.W., HART, I.M. & PATTERSON, D. (1991). A gene correcting the defect in the CHO mutant Ade$^-$H deficient in a branch point enzyme (Adenylosuccinate Synthetase) of *de novo* purine biosynthesis is located on the long arm of chromosome 1. *Genomics*, **9**, 322.

LAJTHA, L.J. & VANE, J.R. (1958). Dependance of bone marrow cells on liver for a purine supply. *Nature*, **182**, 191.

LE HIR, M. & DUBRACH, U.C. (1985). Uphill transport of pyrimidine nucleosides in renal bursh border vesicles. *Plfugers Arch.*, **404**, 238.

LELEIKO, N.S., BRONSTEIN, A.D., BALIGA, B.S. & MUNRO, H.N. (1983). *De novo* purine synthesis in the small intestine. *J. Pediatr. Gastroenterol.*, **2**, 313–319.

LENNARD, L. & LILLEYMAN, J.S. (1987). Are children with lymphoblastic leukemia given enough 6-mercaptopurine? *Lancet*, **ii**, 785.

LENNARD, L. & LILLEYMAN, J.S., (1989). Variable mercaptopurine metabolism and treatment outcome in childhood lymphoblastic leukemia. *J. Clin. Oncol.*, **7**, 1816.

LI, G.C. (1987). Heat shock proteins: role in thermotolerance, drug resistance and relationship to DNA topoisomerases. *Natl Cancer Inst. Monograph*, **4**, 99.

LOCK, L.F., MELTON, D.W., CASKEY, C.T. & MARTIN, G.R. (1986). Methylation of the mouse HPRT gene differs on the active and inactive X Chromosome. *Mol. Cell Biol.*, **6**, 914.

LOW, E. & KUFE, D. (1981). Synergistic effects of inhibitors of *de novo* pyrimidine synthesis avicin and N(phosphonoacetyl)-L-aspartic acid. *Cancer Res.*, **41**, 3419.

LUTHJE, J. (1989). Extracellular adenine compounds, red blood cells and haemostasis: facts and hypotheses. *Blut*, **59**, 367.

MACKINNON, A.M. & DELLER, D.J. (1973). Purine nucleotide biosynthesis in gastrointestinal mucosa. *Biochim. Biophys. Acta*, **319**, 1.

MCDONALD, J.A. & KELLY, W.N. (1971). Lesch-Nyhan syndrome: altered kinetic properties of mutant enzyme. *Science*, **171**, 689.

MEINKOTH, J., KILLARY, A.M., FOURNIER, R.E.K. & WAHL, G.M. (1987). Unstable and stable CAD gene amplification: importance of flanking sequences and nuclear environment in gene amplification. *Mol. Cell Biol.*, **7**, 1415.

MOSCOW, J.A. & COWAN, K.H. (1988). Multidrug resistance. *J. Natl Cancer Inst.*, **80**, 14.

MOSCOW, J.A., TOWNSEND, A.L., GOLDSMITH, M.E. & 6 others (1988). Isolation of the human anionic glutathione S transferase and relation of its gene expression to estrogen receptor content in primary breast cancer. *Proc. Natl Acad. Sci. USA*, **85**, 6518.

MOYER, J.D. & HANDSCHUMACHER, R.E. (1979). Selective inhibition of pyrimidine synthesis and depletion of nucleotide pools by N-(phosphonoacetyl)-L-aspartate. *Cancer Res.*, **39**, 3089.

MOYER, J.D., OLIVER, J.T. & HANDSCHUMACHER, R.E. (1981). Salvage of circulating pyrimidine nucleosides in the rat. *Cancer Res.*, **41**, 905.

NALBANTOGLU, J. & MEUTH, M. (1986). DNA amplification-deletion in a spontaneous mutation of the hamster aprt locus: structure and sequence of the novel joint. *Nucl. Acid. Res.*, **14**, 8361.

NATSUMEDA, Y., PRAJDA, N., DONOHUE, J.P., GLOVER, J.L. & WEBER, G. (1984). Enzymic capacities of purine *de novo* and salvage pathways for nucleotide synthesis in normal and neoplastic tissues. *Cancer Res.*, **44**, 2475.

NATSUMEDA, Y., IKEGAMI, T., OLAH, E. & WEBER, G. (1989). Significance of purine salvage in circumventing the action of antimetabolites in rat hepatoma cells. *Cancer Res.*, **49**, 88.

NELSON, J.A. & DRAKE, S. (1984). Potentiation of methotrexate toxicity by dipyridamole. *Cancer Res.*, **44**, 2493.

OTTO, E., MCCORD, S. & TLSTY, T.D. (1989). Increased incidence of CAD gene amplification in tumourigenic rat lines as an indicator of genomic instability of neoplastic cells. *J. Biol. Chem.*, **264**, 3390.

PASTAN, I.H. & GOTTESMAN, M.M. (1988). Molecular biology of multidrug resistance in human cells. *Important Adv. Oncol.*, De Vita, V.T., Hellman, S. & Rosenberg, S.A. (eds), p. 3. J.P. Lippincott: Philadelphia.

PATERSON, A.R.P., JAKOBS, E.S., NG, C.Y.C., ODEGARD, R.D. & ADJEI, A.A. (1987). Nucleoside transport inhibition *in vitro* and *in vivo*. In *Topics and Perspectives in Adenosine Research*, Gerlach, E. & Becker, B.F., (eds), pp. 89–101. Springer-Verlag: Berlin, Heidelberg.

PIAFSKY, K.M. & BORGA, O. (1977). Plasma protein binding of basic drugs. II. Importance of $\alpha_1$-acid glycoprotein for interindividual variation. *Clin. Pharmacol. Therapeut.*, **22**, 545.

PIETERS, R., HUISMANS, D.R. & VEERMAN, A.J.P. (1987). Are children with lymphoblastic leukemia resistant to 6-mercaptopurine because of 5′ nucleotidase? *Lancet*, **ii**, 1471.

PIETERS, A. & VEERMAN, A.J.P. (1988). The role of 5′ nucleotidase in therapy-resistances of childhood leukemia. *Med. Hypoth.*, **27**, 77.

PLAGEMANN, P.G.W. & BEHRENS, M. (1976). Inhibition of *de novo* pyrimidine nucleotide and DNA synthesis on growth of cultured Novikoff rat hepatoma cells and other cell lines by pyazofurin (NSC 14095). *Cancer Res.*, **36**, 3807.

PLAGEMANN, P.G.W., MARZ, R., WOHLHUETER, R.M., GRAFF, J.C. & ZYLKA, J.M. (1981). Facilitated transport of 6-mercaptopurine and 6-thioguanine and non-mediated permeation of 8-azaguanine in Novikoff rat hepatoma cells and relationship to intracellular phosphoribosylation. *Biochim. Biophys. Acta*, **647**, 49.

PLAGEMANN, P.G.W., WOHLEUTER, R.M. & WOFFENDIN, C. (1988). Nucleoside and nucleobase transport in animal cells. *Biochim. Biophys. Acta*, **947**, 405.

PLAGEMANN, P.G.W. & WOFFENDIN, C. (1989). $Na^+$-dependent and -independent transport of uridine and its phosphorylation in mouse spleen cells. *Biochim. Biophys. Acta*, **981**, 315.

PRUS, K.L., AVERETT, D.R. & ZIMMERMAN, T.P. (1990). Transport and metabolism of 9-β-D-Arabinofuranosylguanine in a human T-lymphoblastoid cell line. Nitrobenzylthioinosine sensitive and insensitive influx. *Cancer Res.*, **50**, 1817.

PUCHALSKI, R.B. & FAHL, W.E. (1990). Expression of recombinant glutathione S-transferase II γa or γb confers resistance to alkylating agents. *Proc. Natl Acad. Sci. USA*, **87**, 2443.

REID, L., GREGG, R.G., SMITHIES, O. & KOLLER, B.H. (1990). Regulatory elements in the introns of human HPRT gene are necessary for its expression in embryonic stem cells. *Proc. Natl Acad. Sci. USA*, **87**, 4299.

RIGGS, A.D. & JONES, P.A. (1983). 5-methylcytosine, gene regulation and cancer. *Adv. Cancer Res.*, **40**, 1.

ROSE, K.M. (1988). DNA topoisomerase as targets for chemotherapy. *FASEB J.*, **2**, 2474.

SAVIANO, D.A. & CLIFFORD, A.J. (1981). Adenine: the precursor of nucleic acids in intestinal cells which are unable to synthesise purines by the *de novo* pathway. *J. Nutr.*, **111**, 1816.

SCAVANNEC, J.D., MARANICHINI, J.A., GASTAUT, Y., CARCASOONE, J. & CAILLA, H.L. (1982). Purine and pyrimidine ribonucleoside monophosphate patterns of peripheral blood and bone marrow cellsin human acute leukemias. *Cancer Res.*, **42**, 1326.

SCHIMKE, R.T., ALT, F.W., KELLEMS, R.E., KAUFMAN, R.J. & BERTINO, J.R. (1977). Amplification of dihydrofolate reductase genes in methotrexate-resistant cultured mouse cells. *Cold Spring Harbor Symp. Quant. Biol.*, **42**, 649.

SCHIMKE, R.T. (1984). Gene amplification, drug resistance and cancer. *Cancer Res.*, **44**, 1735.

SCHWENK, M., HEGAZY, E. & LOPEZ EL PINO, V. (1984). Uridine uptake by isolated intestinal epithelial cells of guinea pig. *Biochim. Biophys. Acta*, **805**, 370.

SINKELER, S., JOESTEN, E., WEVERS, R., BINKHORST, R. & OEI, L. (1986). Skeletal muscle adenosine, inosine and hypoxanthine release following ischeamic forearm exercise in myoadenylate deaminase deficiency and McArdle's disease. *Adv. Exptl Med. Biol.*, **195B**, 517.

SOBRESO, A.F., MOIR, R.D., BERTINO, J.R. & HANDSCHMACHER (1985). Defective facilitated diffusion of nucleosides as a primary mechanism of resistance to 5-flouro-2-deoxyuridine in HT8 human carcinoma line. *Cancer Res.*, **45**, 3155.

SOLLERI, A., TORSSELL, L., OWAL, A., EDLUND, A. & LAGERKRANSER, M. (1987). Levels and cardiovascular effects of adenosine in humans. In *Topics and Perspectives in Adenosine Research*, Gerlach, E. & Becker, B.F. (eds), pp. 599–613. Springer-Verlag: Berlin, Heidelberg.

SOWEMIMO-COKER, S.O., KOVACS, I.B., PICKLES, H., HEDGES, A. & TURNER, P. (1983). Dipyridamole increases red cell deformability. *Br. J. Pharmacol.*, **16**, 423.

SPYCHALA, J., MADRID-MARINA, Y. & FOX, J.H. (1988). High $K_m$ soluble 5′ nucleotidase from human placenta. Properties and allosteric regulation by IMP and AMP. *J. Biol. Chem.*, **263**, 18759.

SZEBENI, J., WAHL, S.M., POPOVIC, M. & 6 others (1989). Dipyridamole potentiates the inhibition by 3′-azido-3′deoxythymidine and other dideoxynucleosides of human immunodeficiency virus replication in monocyte-macrophages. *Proc. Natl Acad. Sci. USA*, **86**, 3842.

THOMPSON, L.F. (1986). Ecto-5′ nucleotidase can use IMP to provide the total purine requirements of mitogen-stimulated human T cells and rapidly dividing human B lymphoblasts. *Adv. Exptl Med. Biol.*, **1958**, 467.

TURNER, D.R., MORLEY, A.A., HALIANDROS, M., KUTLACA, R. & SANDERSON, B.J. (1985). *In vivo* somatic mutations in human lymphocytes frequently result from major genetic alterations. *Nature*, **315**, 343.

VAN DIGGELEN, O.P., DONAHUE T.F. & SHIN, I. (1979). Basis for differential cellular sensitivity to 8-azaguanine and 6-thioguanine. *J. Cell. Phys.*, **98**, 59.

VAN LOON, J.A. & WEINSHILBOUM, R.M. (1982). Thiopurine methyltransferase biochemical genetics: human lymphocyte activity. *Biochem. Genet.*, **20**, 637.

VEERMAN, A.J.P., HOGEMAN, P.H.G., VAN ZANTWIJK, C.H. & BEZEMER, P.D. (1985). Prognostic value of 5′ nucleotidase in acute lymphoblastic leukemia with common-ALL phenotype. *Leukemia Res.*, **9**, 1227.

VERSCHUERUEN, H., WILDEMAUWE, C. & LAREBEKE, N. (1983). Effects of dipyridamole (Persantin R) on the morphology and motility of mouse embryo cells. *Cell Biol. Int. Rep.*, **7**, 263.

WAHL, G.M., PADGETT, R.A. & STARK, G.R. (1979). Gene amplification causes over production of the first three enzymes of UMP synthesis in N-(phosphonoacetyl)-L-aspartate-resistant hamster cells. *J. Biol. Chem.*, **254**, 8679.

WALSH, M.J., SANCHEZ-POZO, A. & LELEIKO, N.S. (1990). A regulatory element is characterised by purine mediated and cell-type specific gene transcription. *Mol. Cell Biol.*, **10**, 4356.

WEBER, G. (1983). Biochemical strategy of cancer cells and the design of chemotherapy: G.H.A. Clowes Memorial Lecture. *Cancer Res.*, **43**, 3466.

WEBER, G., JAYARAM, H.N., LAPIS, E. & 5 others (1988). Enzyme-pattern-targeted chemotherapy with tiazofurin and allopurinol in human leukemia. *Adv. in Enzyme Regulation*, **27**, 405.

WEINSHILBOUM, R.M. & SLADEK, S.L. (1980). Mercaptopurine pharmacogenetics: monogenic inheritance of erythrocyte thiopurine methyltransferase activity. *Am. J. Hum. Genet.*, **32**, 651.

WEISMAN, G.A., LUSTIG, K.D., LANE, E., HUANG, N., BELZER, I. & FRIEDBERG, I. (1988). Growth inhibition of transformed mouse fibroblasts by adenine nucleotides occurs via generation of extracellular adenosine. *J. Biol. Chem.*, **263**, 12367.

WHITEHOUSE, J.M. (1985). Concepts of drug resistance: clinical setting. In *Antitumour Drug Resistance*, Fox, B.W. & Fox, M. (eds), pp. 3–21. Springer Verlag: Berlin, Heidelberg, London, New York.

WILLIAMS, A.M. (1962). Nucleic acid metabolism in leukemic human leukocytes 1. *In vitro* incorporation by leukocytes from chronic granulocytic leukemia. *Cancer Res.*, **22**, 314.

WOHLHUETER, R.M., MCIVOR, R.S. & PLAGEMANN, P.G.W. (1980). Facilitated transport or uracil and 5-florouracil and permeation of orotic acid into cultured mammalian cells. *J. Cell. Physiol.*, **104**, 309.

WONG, W.E. & HOWELL, S.B. (1984). Hypoxanthine concentrations in normal subjects and patients with solid tumours and leukemia. *Cancer Res.*, **44**, 3144.

WOOD, A.W., BECKER, M.A. & SEEGMILLER, J.E. (1973). Purine nucleotide synthesis in lymphoblasts cultured from normal subjects and a patient with Lesch-Nyhan syndrome. *Biochem. Genetics*, **9**, 261.

WRIGHT, J.A., SMITH, H.S., WATT, F.M., HANCOOK, M.C., HUDSON, D.L. & STARK, G.R. (1990). DNA amplification is rare in normal human cells. *Proc. Natl Acad. Sci. USA*, **87**, 1791.

YEN, P.H., MOHANDAS, T. & SHARPIO, L.J. (1986). Stability of DNA methylation of the human phosphoribosyl transferase gene. *Somatic Cell Mol. Genet.*, **12**, 153.

ZIEG, J., CLAYTON, C.E., ARDESHIR, F., GIULETTO, E., SWYRYD, A. & STARK, G.R. (1983). Properties of single-step mutants of Syrian hamster cell-lines resistant to N-(phosphonacetyl)-L-aspartate. *Mol. Cell. Biol.*, **3**, 2089.

ZIMM, S., COLLINS, J.M., RICCARDI, R. & 4 others (1984). Variable bioavailability of oral mercaptopurine. *New Engl. J. Med.*, **308**, 1005.

ZIM, S., ETTINGER, L.J., HOLCENBERG, J.A. & 8 others (1985). Phase 1 and clinical pharmacological study of mercaptopurine administered as a prolonged intravenous infusion. *Cancer Res.*, **45**, 1869.